# Climate connectivity of the bobcat in the Great Lakes region

**DOI:** 10.1002/ece3.6049

**Published:** 2020-01-28

**Authors:** Robby R. Marrotte, Jeff Bowman, Paul J. Wilson

**Affiliations:** ^1^ Environmental & Life Sciences Graduate Program Trent University Peterborough ON Canada; ^2^ Wildlife Research & Monitoring Section Ontario Ministry of Natural Resources & Forestry Peterborough ON Canada; ^3^ Biology Department Trent University Peterborough ON Canada

**Keywords:** Bobcat, functional connectivity, gene flow, Great Lakes region, landscape genetics, *Lynx rufus*, range expansion

## Abstract

The Great Lakes and the St. Lawrence River are imposing barriers for wildlife, and the additive effect of urban and agricultural development that dominates the lower Great Lakes region likely further reduces functional connectivity for many terrestrial species. As the climate warms, species will need to track climate across these barriers. It is important therefore to investigate land cover and bioclimatic hypotheses that may explain the northward expansion of species through the Great Lakes. We investigated the functional connectivity of a vagile generalist, the bobcat, as a representative generalist forest species common to the region. We genotyped tissue samples collected across the region at 14 microsatellite loci and compared different landscape hypotheses that might explain the observed gene flow or functional connectivity. We found that the Great Lakes and the additive influence of forest stands with either low or high canopy cover and deep lake‐effect snow have disrupted gene flow, whereas intermediate forest cover has facilitated gene flow. Functional connectivity in southern Ontario is relatively low and was limited in part by the low amount of forest cover. Pathways across the Great Lakes were through the Niagara region and through the Lower Peninsula of Michigan over the Straits of Mackinac and the St. Marys River. These pathways are important routes for bobcat range expansion north of the Great Lakes and are also likely pathways that many other mobile habitat generalists must navigate to track the changing climate. The extent to which species can navigate these routes will be important for determining the future biodiversity of areas north of the Great Lakes.

## Graphical abstract

The range of the bobcat has expanded northward in the Great Lakes region in North America. We compared expectations from different models of expansion with genetic observations and then mapped observed pathways north through the Great Lakes region. We found that many of the routes used by this vagile generalist crossed large water bodies and highly modified areas that are not likely to be navigable by less mobile species while they track the changing climate.

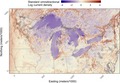

## 1 INTRODUCTION

Climate is a dominant driver of species range expansion and contraction (Davis & Shaw, 2001; Huntley, 1999; Woodward & Williams, 1987); as a result, the warming climate will inevitably shift the range of many species (Bellard, Bertelsmeier, Leadley, Thuiller, & Courchamp, 2012; Thomas et al., 2004). In fact, we have already witnessed climate‐induced range shifts of numerous species (Chen, Hill, Ohlemüller, Roy, & Thomas, 2011; Hickling, Roy, Hill, Fox, & Thomas, 2006; Laliberte & Ripple, 2004; Parmesan, 2006). Many species can adapt and persist within its original range (Bellard et al., 2012; Durant, Hjermann, Ottersen, & Stenseth, 2007; Gardner, Heinsohn, & Joseph, 2009), but other species that face geographic barriers and cannot keep pace with the velocity of climate change might risk extinction (Thomas et al., 2004).

The ability of a species to track the changing climate by shifting its range depends in part on its niche requirements, habitat availability, and connectivity (Leroux et al., 2013; Robillard, Coristine, Soares, & Kerr, 2015). If a species is not able to adapt to local changes, its persistence depends on the degree to which the landscape promotes or hinders the dispersal of individuals (Fahrig & Merriam, 1985; Fahrig & Paloheimo, 1988; Taylor, Fahrig, Henein, & Merriam, 2006). A species’ dispersal capability might depend on its physiological limitations (Travis et al., 2013), its demography (Clark, Lewis, & Horvath, 2017), or its behavior (Ehrlich, 1961; Pusey, 1987; Warren et al., 2001). Large physical barriers such as mountains, oceans, lakes, and rivers can impede movement of individuals, disrupting gene flow (Grant & Grant, 2009; Koen, Bowman, & Wilson, 2015; Stebbins, 1949; Steeves, Anderson, McNally, Kim, & Friesen, 2003).

Many highly mobile species might indeed be able to track contemporary climate change across natural landforms, but the addition of cities, highways, roads, and agricultural crops can potentially hinder mobility in an additive fashion (Epps et al., 2005; Riley et al., 2006; Robillard et al., 2015). Furthermore, suitable habitat might be found several hundreds of kilometers north of a species’ current range, but the environmental characteristics of the interstitial landscape could be well outside of its niche (Early & Sax, 2011). In such a case, the environment might impede or block dispersal and the colonization of newly available habitat (McRae, 2006; Wang & Bradburd, 2014). Ultimately, future biodiversity across the globe will depend on the ability of species to rapidly disperse throughout continental‐scale habitat networks to keep up with changing environmental conditions. Many animals will need to migrate across human‐dominated and highly modified landscapes to colonize new habitats. It is therefore necessary to understand the effect that natural and anthropogenic barriers have on a species dispersal ability and to understand how these barriers influence connectivity across entire regions.

Continent‐wide range expansion pathways are usually inferred from stationary biological information (estimates of dispersal distance and niche requirements) and rarely from observed patterns of movement (Bagchi et al., 2018; Krosby, Theobald, Norheim, & McRae, 2018; Lawler, Ruesch, Olden, & Mcrae, 2013; McGuire, Lawler, Lawler, McRae, Nuñez, & Theobald, 2016; Zhang et al., 2019). Past population dynamics are imprinted in the genes of species (Fordham, Brook, Moritz, & Nogués‐Bravo, 2014). This information can be used to infer past patterns of movements of individuals and their genes across the landscape (Holderegger & Wagner, 2008). Such patterns have been observed in many species in the context of range change (Greenhorn, Bowman, & Wilson, 2018; Koen, Bowman, Murray, & Wilson, 2014; Sivyer, Morgan‐Richards, Koot, & Trewick, 2018; Zakharov & Hellmann, 2008).

The Great Lakes are visibly the largest natural barrier to terrestrial species migration in eastern North America. Currently, we do not know for many species to what extent the Great Lakes have influenced movement and consequently gene flow, although the impacts are likely profound; much smaller barriers such as canals, highways, mountains, rivers, roads, sea lochs, and urban development have been shown to restrict the gene flow of many terrestrial vagile species (Blanchong et al., 2008; Coulon et al., 2006; Cushman & Lewis, 2010; Epps et al., 2005; Koen et al., 2015; Kuehn et al., 2007; Pérez‐Espona et al., 2008; Proctor, McLellan, Strobeck, & Barclay, 2005; Riley et al., 2006; Robinson, Samuel, Lopez, & Shelton, 2012; Robinson, Samuel, Rolley, & Shelton, 2013; Vander Wal, Paquet, & Andraés, 2012). Unfortunately, the additive influence of anthropogenic disturbance between and within the vicinity of the Great Lakes will likely further restrict gene flow through these large natural barriers for many species.

The bobcat (*Lynx rufus*) is the most widely distributed feline species in North America, and there is evidence that it was more abundant across the continent before European colonization and during the Pleistocene (Deems & Pursley, 1983; Graham & Lundelius, 2010; Lariviere & Walton, 1997). It is generally thought that intensive trapping and land clearing led to the extirpation of the species in the midwestern United States and many parts of the Great Lakes region and this may also have caused the apparent absence of the species in many areas of the Midwest United States (rode Deems & Pursley, 1978; Deems & Pursley, 1983; Vos, 1964; Woolf, Nielsen, & Gibbs‐Kieninger, 2000).

In recent decades, bobcat sightings, road kills, and individuals incidentally harvested by trappers have become more common in the region (Marrotte, Bowman, & Morin In Review; Roberts & Crimmins, 2010; Woolf & Hubert, 1998). There is evidence that bobcat populations are spreading into areas where they were thought to be absent (Linde, Roberts, Gosselink, & Clark, 2012; Woolf & Hubert, 1998). For example, incidental trapper records indicate that the bobcat range is expanding north into northern Ontario, Canada, from Minnesota and from the Upper Peninsula of Michigan (UPM).

Bobcats are not spreading to the same extent into southern Ontario however, even though they once inhabited this landscape (de Vos, 1964). Landscape configuration could be playing an important role in structuring the recolonization of the bobcat in the Great Lakes region. For instance, the Great Lakes and the St. Lawrence River are imposing barriers to movement for wildlife. In addition, urban development and agricultural development dominate southern Ontario and may be impeding bobcats from colonizing this range frontier, over and above the barrier effect of the Great Lakes. There is evidence, however, that the bobcat can cope with an anthropogenic environment (Lee et al., 2012; Riley et al., 2003; Tigas, Vuren, & Sauvajot, 2002; Woolf et al., 2000). For example, in Illinois, the bobcat occupies landscapes with intensive agriculture (Woolf et al., 2000). It also seems capable of occupying areas surrounded by transportation infrastructure and urban development (Lee et al., 2012; Riley et al., 2003; Tigas et al., 2002). However, urban land cover and major highways have caused reduced gene flow in bobcat populations in California (Kozakiewicz et al., 2019; Lee et al., 2012).

Snow is also considered by some to be a limiting factor to bobcat expansion north of its range, as many researchers have suggested that the species has high foot loading and cannot efficiently travel and hunt in deep snow (Hoving, Joseph, & Krohn, 2003; Marston, 1942; McCord, 1974; Parker, Maxwell, Morton, & Smith, 1983). For example, McCord (1974) found that the bobcat had a difficult time traveling through areas that had a sinking depth exceeding 15 cm. Also, Parker et al. (1983) suggested that the reason the bobcat did not invade the highlands of Cape Breton was because of the deeper snow. In addition, snow clearing and compaction near human settlements may mediate the influence of snow on colonization and may promote bobcats from occupying areas north of their range (Marrotte et al., In Review).

We investigated several land cover and bioclimatic hypotheses that may explain the northward expansion of the bobcat throughout the Great Lakes region in North America, because any restrictions imposed on a highly mobile species would likely be even more perilous for less vagile species. We hypothesized that barriers to northward expansion would hinder gene flow. We considered that there are 3 scenarios that may describe range expansion in the Great Lakes region:

- *H*_0_: Panmixia: Natural and anthropogenic barriers have no effect on gene flow; thus, individuals are panmictic.
- *H*_1_: Isolation by distance (IBD): Natural and anthropogenic barriers have no effect on gene flow, but gene flow decays over geographic distance.
- *H*_2_: Isolation by resistance (IBR): Natural and anthropogenic barriers constrict gene flow; thus, flow percolates through land bridges between the lakes.

**Figure 1:**
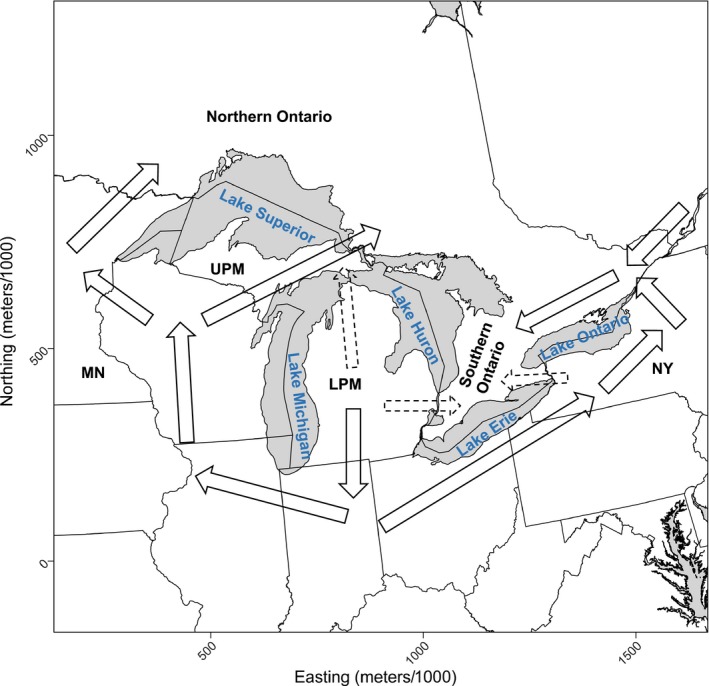
Predictions of northward expansion of a vagile habitat generalist across the Great Lakes region in Canada and the United States. MN, Minnesota, USA; NY, New York, USA; LPM, Lower Peninsula of Michigan, USA; UPM, Upper Peninsula of Michigan, USA. Spatial layers for administrative boundaries were gathered from the Database of Global Administrative Areas

The bobcat is an ideal study species to test our hypotheses of range expansion in the context of anthropogenic change, because it is a vagile habitat generalist that is currently expanding its range and demonstrates some limitations to human disturbance and climate. We predicted that gene flow of the bobcat is obstructed naturally by the Great Lakes and deep snow but also hindered by low forest cover and by the transportation infrastructure. This model most closely follows the isolation‐by‐resistance hypothesis (*H*_2_) previously described. Therefore, we predicted that gene flow is constricted through certain pathways that connect individuals throughout the region (Figure 1). We predicted that gene flow in southern Ontario originated mostly from the east from the province of Quebec and New York State, since flow is limited through the Lower Peninsula of Michigan (LPM) and between Lake Ontario and Erie, because of the high road density and low forest cover of these regions. On the other hand, northern Ontario is connected to the south by a more natural landscape with high forest cover and less human disturbance. Consequently, gene flow to northern Ontario is facilitated by the Upper Peninsula of Michigan (UPM) and the largely forested area to the west of Lake Superior. Our rationale is that range expansion in this region is restricted by the additive effect of natural and anthropogenic barriers. Gene flow should be constricted and forced to pass through land in between and around the Great Lakes, while deep snow should reduce the capability of flow northwards and cause gene flow to deviate around areas that receive high annual snowfall caused by the lake effect (Norton & Bolsenga, 1993). The upper Great Lakes are periodically hit by frequent lake‐effect snowfall or snow squalls with over 15 cm of snow accumulation in a single day (Baijnath‐Rodino & Duguay, 2018). In addition, gene flow should be hindered by agricultural areas with low cover such as the corn belt areas of the Midwest and areas with high density of roads such as urban areas.

## 2 MATERIALS AND METHODS

### 2.1 Study area

**Figure 2:**
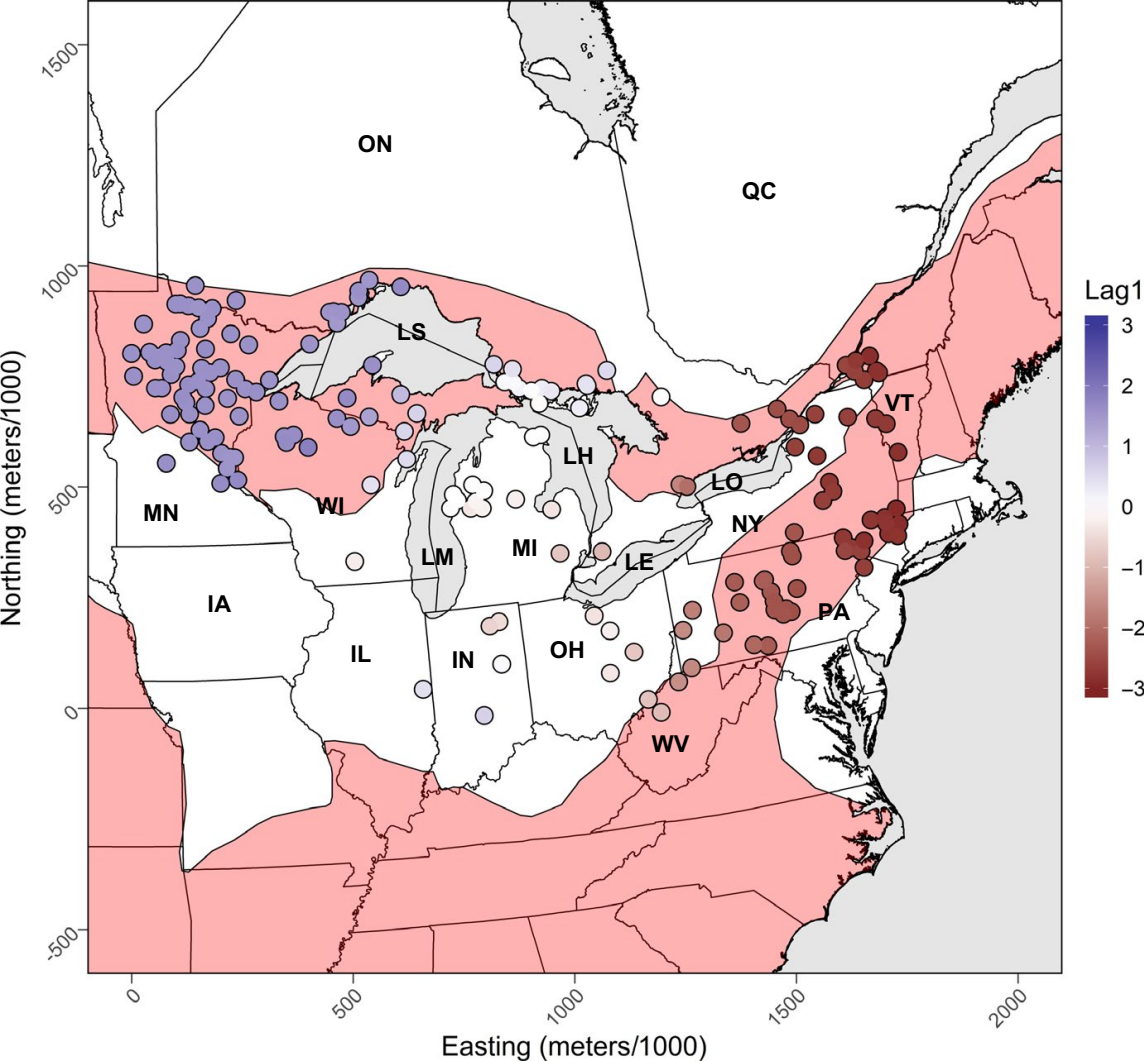
Location of 240 bobcat (*Lynx rufus*) fur samples collected from a variety of sources between 2012 and 2017 across the Great Lakes region in Canada and the United States. The shaded area is the consensus bobcat range according to the IUCN and the Nature Conservancy. Lag1 is the first axis from a spatial principal component analysis on the alleles scores of bobcats. It represents the only significant major spatial variation across bobcats in the Great Lakes region. Labels are as follows: ON, Ontario, Canada; QC, Quebec, Canada; MN, Minnesota, USA; WI, Wisconsin, USA; MI, Michigan, USA; NY, New York, USA; VT, Vermont, USA; IA, Iowa, USA; IN, Indiana, USA; OH, Ohio, USA; PA, Pennsylvania, USA; WV, West Virginia; LS, Lake Superior, LM, Lake Michigan; LH, Lake Huron; LE, Lake Erie; LO, Lake Ontario

From 2012 to the end of 2017, we collected bobcat pelt samples from the North American Fur Auction (NAFA), Ontario Ministry of Natural Resources and Forestry, researchers, and trappers (Figure 2). We sampled bobcat pelts found within an area around the Great Lakes defined by the maximum dispersal distance of the bobcat of 300 km (Johnson, Walker, & Hudson, 2010). We sampled on both sides of the international border between Canada and the United States.

### 2.2 Genetic analysis

We followed the laboratory protocols and scoring methodology of Koen, Bowman, Murray, et al. (2014) and Row et al. (2012) and genotyped bobcat samples at 14 microsatellite loci (Fca031, Fca035, Fca043, Fca077, Fca090, Fca096, Fca441, Fca391, Fca559, Lc106, Lc109, Lc110, Lc111, and Lc118). We removed individuals that had any missing loci and individuals that were not correctly georeferenced.

We then explored the spatial structure of these data using a spatial principal component analysis (sPCA; Jombart, Devillard, Dufour, & Pontier, 2008) and tested for patterns of spatial autocorrelation. We used a distance‐based nearest neighbor approach, where individuals within 300 km were assumed to be neighbors, since bobcat have been observed to disperse up to 288 km from their natal range (Johnson et al., 2010; Knick & Bailey, 2006). We also tested different neighborhood approaches and found similar spatial patterns. We used a Monte Carlo test to determine whether there were any global spatial structures (broadscale clusters or clines) or local spatial structures (fine‐scale disparity among neighbors) worth investigating (Jombart, 2008). We permuted the alleles scores 9,999 times to test the significance of the spatial structure. If there was no spatial structure, then the panmixia hypothesis (*H*_0_) would be concluded, because our isolation‐by‐distance (*H*_1_) and isolation‐by‐resistance (*H*_2_) hypotheses were inherently spatial. After exploring the spatial structure, we then used the proportion of shared alleles as a metric of genetic similarity between individuals and tested our spatial hypotheses.

### 2.3 Gene flow covariates

**Figure 3:**
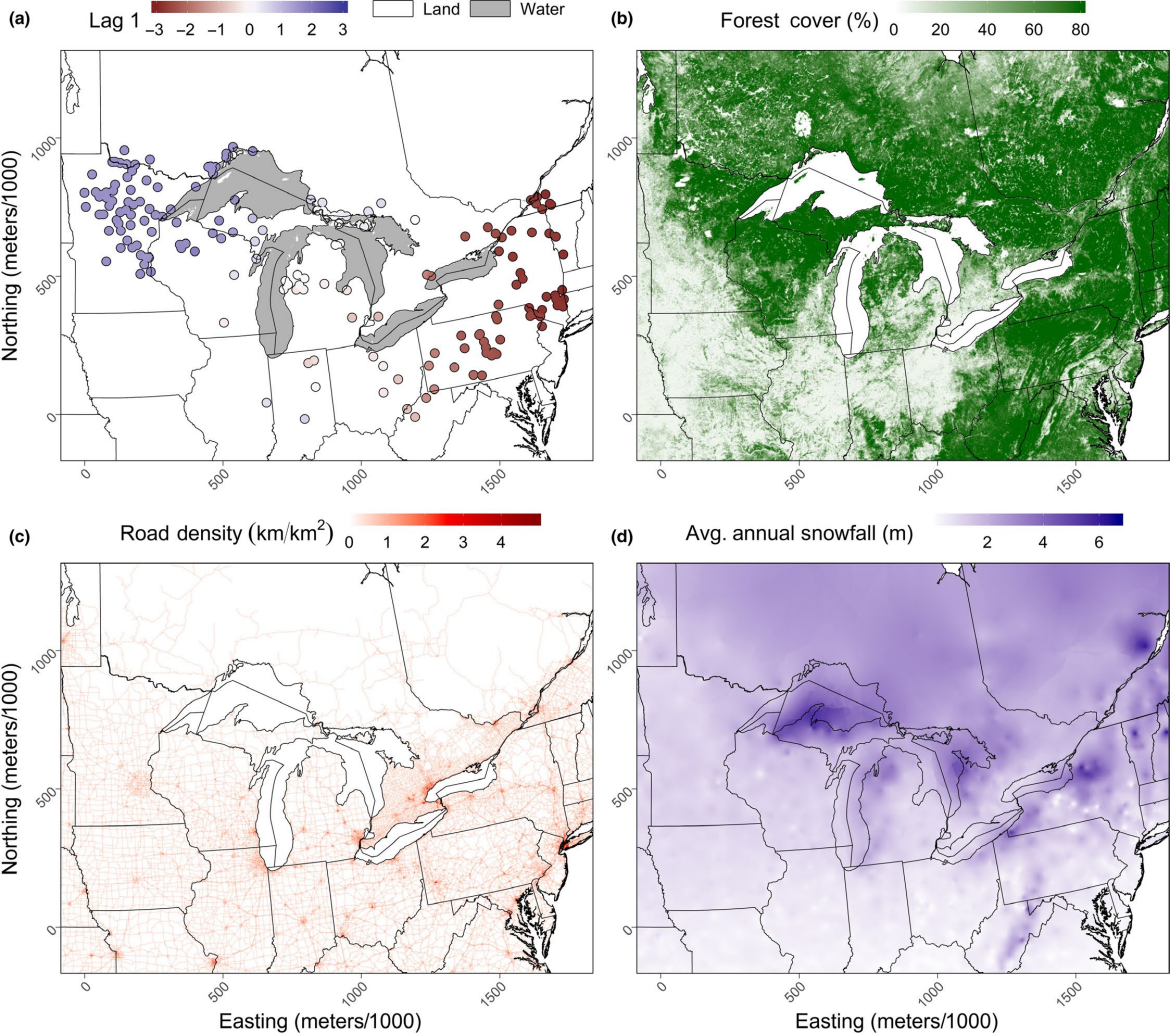
Landscape maps used to test isolation‐by‐resistance hypotheses of bobcat gene flow across the Great Lakes region. (a) Great Lake land barrier, (b) forest cover, (c) road density, and (d) annual snowfall. In total, 8 isolation‐by‐resistance models were tested and included only combinations of the Great Lakes with all other three maps. We also compared these models to a null model of panmixia (*H*_0_) and isolation by distance (*H*_1_)

We built 4 different landscape maps, which we thought could explain bobcat gene flow in the Great Lakes region and which would allow us to test our isolation‐by‐resistance hypothesis (Figure 3). To establish the spatial extent of our analysis, we used the minimum convex hull that contained the Great Lakes with a 400 km buffer to leave 100 km between the edge of the map and any bobcat samples (Koen, Garroway, Wilson, & Bowman, 2010). The Great Lakes spatial layer we used to create this boundary and the St. Lawrence river layer that we later used were gathered from the Lakes and Rivers, 2009, spatial layers freely available by the Commission for Environmental Cooperation (CEC; cec.org).

To create our Great Lakes landscape layer, we assigned values of 1 to areas where there was either the Great Lakes or the St. Lawrence and the value 2 to the land (Figure 3a). 

We used the forest cover layer provided by the University of Maryland, which has a continuous forest cover field where each pixel has an assigned value that represented the percentage of tree cover as the data source for our second map (glcf.umd.edu/data/treecover; DeFries, Hansen, Townshend, Janetos, & Loveland, 2000). This forest cover layer extended across North America at a resolution of 1 km^2^ with values that ranged from 10% to 80% forest cover. However, there were values of 254 and 255, which represented areas that were nonvegetated and areas where tree cover was less than 10%. We assigned a value of 1 to areas that were nonvegetated and a value of 5% to areas that were less than 10% vegetated (Figure 3b). We were not able to find tree cover at such a fine resolution that matched the temporal resolution of our bobcat data, but there has not been much recent forest loss in our study area; from 2000 to 2017, the largest forest loss was in Minnesota, which had a decrease in forest cover by 6.9% (globalforestwatch.org).

We used the freely available road layer provided by Natural Earth (naturalearthdata.com) to create our next landscape. We calculated the road density within a radius of 1 km from the center of a pixel with a resolution of 1 km (Figure 3c).

For our snow layer, we gathered annual data from the Global Historical Climatology Network (GHCN; http://ncdc.noaa.gov/snow-and-ice), which provides data tables for climate stations found across the world. We chose stations found within Canada and the United States that had more than 15 years of data between 1980 and 2011 and calculated the annual snowfall mean of each climate station. We then interpolated these data using ordinary spherical kriging (Figure 3d).

All surfaces were aggregated using the mean for continuous surfaces and the mode for discreet surfaces to a resolution of 2 km to reduce computation time. We masked out the ocean using a landform layer also provided by Natural Earth. All our layers were projected to North America Lambert Conformal Conic (https://epsg.io/102009).

### 2.4 Statistical framework

Our hypotheses were that bobcat gene flow across the Great Lakes region is panmictic (*H*_0_), a function of distance (*H*_1_), or a function of resistance (*H*_2_). Therefore, we first investigated 2 null models that simply tested for panmixia and isolation by distance, and we then tested 8 isolation‐by‐resistance models that combined landscape features (Table 1). The Great Lakes barrier was present in all 8 landscape models. We did not find it logical to test isolation‐by‐resistance models in the absence of the Great Lakes, because forest cover, road density, and snow cover are additive effects on gene flow and not solitary effects. We reasoned that bobcats cannot inhabit a lake but could occupy an area where there are no forest cover, high road density, and deep snow. Also, these isolation‐by‐resistance models included the influence of isolation by distance, because of the nature of resistance distance (McRae, 2006).

**Table 1:** Summary statistics for 10 landscape model used to explain bobcat gene flow in the Great Lake region

Model	Consensus rank	Average						Rank percentage		
		AICc	*R* ^2^m	*R* ^2^c	Rank	ΔAICc	ω_i_	1st	2nd	3rd
GL* + Forest + Snow	1	**−1419.60**	0.33	**0.66**	**3.84**	**9.21**	0.20	19.22	**22.12**	**16.52**
Isolation by Distance	2	−1414.24	0.11	0.55	4.20	14.57	0.17	18.82	11.51	10.61
GL + Forest	3	−1416.79	0.25	0.62	4.26	12.02	**0.22**	**22.22**	13.11	9.91
GL + Snow	4	−1415.22	0.25	0.62	4.64	13.59	0.15	15.42	10.51	11.01
GL + Roads	5	−1412.28	0.23	0.60	5.36	16.53	0.08	8.41	9.61	6.51
GL + Roads + Snow	6	−1414.00	0.30	0.64	5.31	14.81	0.11	10.21	12.81	11.71
GL	7	−1409.33	0.13	0.56	5.91	19.48	0.04	3.10	8.41	9.81
GL + Forest + Roads	8	−1411.29	0.29	0.63	6.11	17.52	0.02	2.20	6.41	13.91
GL + Forest + Roads + Snow	9	−1406.65	**0.34**	**0.66**	7.32	22.16	0.00	0.20	2.60	5.71
Panmixia	10	−1399.75	0.00	0.49	8.05	29.06	0.01	0.20	2.90	4.30

Note

Consensus rank was determined using the AICc between the 10 models within each 999 set of replicates. Values in bold font are the best value of each metric. All models except the panmixia (*H*_0_) and isolation by distance (*H*_1_) were isolation‐by‐resistance models (*H*_2_).

* Great Lakes.

For each hypothesis, we fit the proportion of shared alleles using generalized linear mixed models with a normal error structure and a “log” link, since we reasoned that the influence of landscape features on gene flow decays exponentially across space. We also found higher variance explained using an exponential model than a linear model. We used a maximum‐likelihood population‐effects covariance structure to account for the nonindependence of the pairwise nature of the data (Clarke, Rothery, & Raybould, 2002). For our isolation‐by ‐resistance models, we were interested in estimating the resistance of the landscape; consequently, we used circuit theory and landscape resistance optimization (Marrotte, Gonzalez, & Millien, 2014; McRae, 2006; Peterman, 2018). We optimized the resistance of each landscape map with the functions provided in the “ResistanceGA” package (Peterman, 2018) but did not modify any genetic algorithm parameters. We used Circuitscape v.5.3.0 (Anantharaman, Hall, Shah, & Edelman, 2019) in the Julia language v.0.6.2 (Bezanson, Karpinski, Shah, & Edelman, 2012) to calculate the effective distance between individuals (Anantharaman et al., 2019). We fit all mixed‐effects models with the package “lme4” (Bates, Maechler, Bolker, & Walker, 2014) in R v.3.5.1 (R Development Core Team, 2018).

We also accounted for uneven sampling intensity, because it could lead to sampling artifacts (Kierepka & Latch, 2016) and eventually spurious conclusions if not overlooked (Balkenhol & Fortin, 2015). Consequently, we resampled the individuals with replacement into 999 sets of individuals that were at least 100 km apart across the study area. We chose a minimum distance of 100 km, because it gave us the ability to homogenize sampling intensity across the study area and gave us a sufficient number of individuals to investigate. In contrast, a larger distance would have left us with less than 30 individuals and a smaller distance would have left us with quite variable sampling intensity across the study area (Figure 2). In addition, resampling gave us the ability to replicate our models on 999 different combinations of data and therefore gave us the ability to measure the consistency of our results. Given the computation time required to optimize the resistance surfaces and the large number of replicates (7,992), we fit our models using several computer clusters (Cedar, Graham and Orca; computecanada.ca).

We finally ranked each model within its set of replicates with AICc. We also calculated the overall average rank, AICc, ΔAICc, and AICcW_t_ to compare each model. We then used a branch‐and‐bound algorithm in R to find the consensus median ranking of all 10 models using the “ConsRank” package (D’Ambrosio, Amodio, & Mazzeo, 2015).

### 2.5 Functional connectivity

By Ohm's law, circuit resistance is reciprocal to current and fundamental work by McRae (2006) demonstrated that current density is proportional to gene flow (McRae, 2006). To help answer our research question concerning how bobcat populations are connected through the Great Lakes region, we created an omnidirectional current density map. We gathered all 999 optimized resistance surfaces of the top landscape model, and for each surface, we standardized the optimized resistance to the mean. We then calculated the average resistance of each pixel to produce a map of standard average resistance (resistance from this point forward). We then used circuit theory in Circuitscape version 4.05 to produce a current density map (Shah & McRae, 2008). We generally followed the methods of Koen, Bowman, Sadowski, and Walpole (2014) to produce an omnidirectional current density map. However, resistance values cannot be negative; therefore, we first scaled the values between 1 and 100. We then regularly placed 100 nodes at the periphery of the map and simulated current passing between all pairs and summed the total current passing through the Great Lakes region. In conjunction with the optimized average standard resistance surface, this current map gave us an idea where current or gene flow was being impeded.

## 3 RESULTS

We used 240 samples after removing those with missing alleles that were not accurately georeferenced or that were outside our delineated study area (Figure 2). The samples were mostly from bobcats harvested between 2011 and 2017 and 4 were not collected from an auction house. The low number of samples within some US states was a result of the status of legal hunting or trapping or the low abundance of the bobcat. For example, in 2016 the state of Illinois opened the bobcat season after 40 years of being closed, so we were only able to collect a single pelt sample from this US state. In Indiana, we were only able to collect 4 bobcat pelt samples, because hunting and trapping has been closed since 1969.

After performing an sPCA on the allele scores, we found 1 significant global pattern (Observation: 0.020, p‐value: 0.001, Figure 2). There was a NE to NW pattern in bobcat alleles scores across the Great Lakes region, where individuals in both northern corners of the Great Lakes region were found at opposite ends of this gradient.

The proportion of shared alleles ranged from 0.07 to 0.63. All 999 sets of samples had on average 37 individuals and ranged from 31 to 43 individuals. Due to the way we bootstrapped our samples, some samples were selected more often than others. On average, individuals were sampled 154 times and this ranged from 2 to 999 times. There were only 3 samples (from Indiana, Illinois, and Ontario) that were sampled in every set. The 3 individuals were sampled each time, because they were isolated in an area more than 100 km from the nearest other individuals. We checked whether the 3 samples might have driven the optimization of the resistance surface, and we found that the resistance values within 100 km of each of these sites were near the average range compared with other areas on the map (0.17, 0.38 and 0.23). Only 1 individual was sampled as little as twice; this sample was in the highly sampled area in western Minnesota.

The composite landscape model that generally ranked first with AICc within its set using the consensus branch‐and‐bound algorithm included the Great Lakes, forest cover, and annual snowfall (Table 1). In addition, this composite landscape model had the lowest average rank, AICc and ΔAICc. The composite model ranked first 19.2%, second 22.1%, and third 16.5% of the time, and had the largest proportion of its replicates in the top 3 ranks, but the Great Lakes and Forest cover model did have more replicates that ranked first (22.2%; Table 1). The marginal *R* ^2^ for the top composite landscape model had a mean of 0.332 [0.079,0.690], while the conditional *R* ^2^ had a mean of 0.655 [0.446,0.881]. All other models did not rank better than the isolation‐by‐distance model, which had an average marginal *R* ^2^ of 0.106 [0,0.362] with a conditional *R* ^2^ of 0.545 [0.334,0.759]. This indicated that gene flow was a function of geographic distance, and additionally, gene flow was restricted by the Great Lakes, forest cover, and snow over our study area.

Summary statistics from the resistance optimization algorithm allowed us to determine how much each covariate contributed to the optimized resistance surface (Peterman, 2018). We found that the contribution of the forest cover was the highest (*µ* = 58.5%), followed by snow (*µ* = 37.8%) and then the Great Lakes barrier (*µ* = 3.7%). The mean slope of this model was −0.079, with only 6 of 999 iterations having a positive slope. Thus, in most cases, the effective resistance was positively correlated with genetic distance.

**Figure 4:**
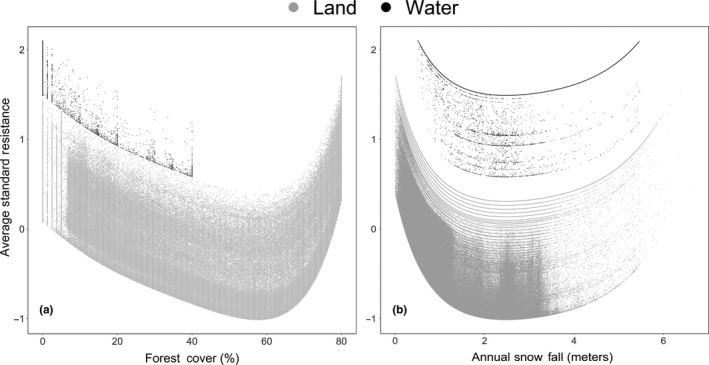
Optimized average standard resistance transformation. (a) Forest cover optimized resistance transformation. In some shorelines, areas forest cover overlapped the Great Lakes layer and values were optimized as if they were the Great Lakes land barrier; therefore, these shore line areas received high average standard resistance simply because of the mismatch between spatial layers. However, in the interior, intermediate forest cover around 60% amplifies gene flow, while low and high forest cover impedes gene flow, but high cover impeded gene flow more over the Great Lakes region. (b) Annual snowfall optimized resistance transformation. Annual snowfall on the lakes was generally transformed to high resistance values compared with land. On land, low annual snowfall impeded gene flow the most, while high annual usually found in lake‐effect areas also impeded gene flow. Like forest cover, intermediate amounts of annual snowfall amplified gene flow over the Great Lakes region

**Figure 5:**
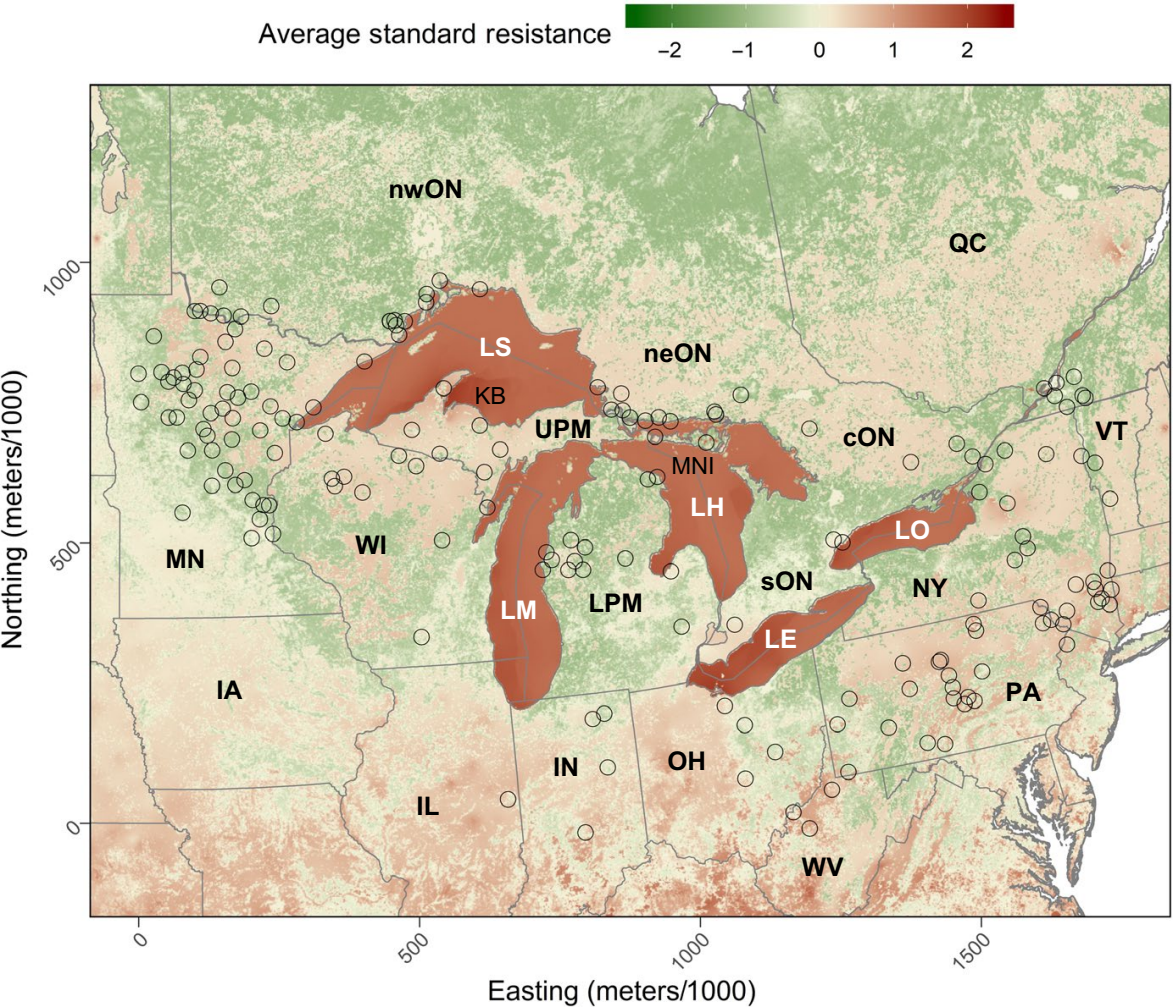
The average standard resistance from 999 replicates of the top model that was fit using resistance surface optimization of a landscape model that included the additive effect of the Great Lake, forest cover, and annual snowfall. These models were fit to the genetic similarity of bobcat samples across the study area. Labels are as follows: nwON, northwestern Ontario, Canada; neON, northeastern Ontario, Canada; cON, central Ontario, Canada; sON, southern Ontario, Canada; QC, Quebec, Canada; MN, Minnesota, USA; WI, Wisconsin, USA; UPM, Upper Peninsula of Michigan, USA; LPM, Lower Peninsula of Michigan, USA; NY, New York, USA; VT, Vermont, USA; IA, Iowa, USA; IN, Indiana, USA; OH, Ohio, USA; PA, Pennsylvania, USA; WV, West Virginia; LS, Lake Superior, LM, Lake Michigan; LH, Lake Huron; LE, Lake Erie; LO, Lake Ontario; KB, Keweenaw Bay; MNI, Manitoulin Island, Ontario, Canada

After scaling all replicates of the optimized surfaces of the best combined landscape model and taking the average through each pixel, we found that the Great Lakes had a higher resistance than other features on the landscape (Figures 4, 5). In fact, the resistance values in the Great Lakes were on average 1.557 [0.579, 2.104] times higher than the rest of the landscape. Generally, areas with low and high forest cover had high resistance values, whereas intermediate values that neared 60% forest cover had the lowest values (Figure 4a). This pattern was also the same for annual snowfall (Figure 4b), areas with low and high annual snowfall had high resistance compared to areas with intermediate annual snowfall that neared 2 meters of annual snowfall.

**Figure 6:**
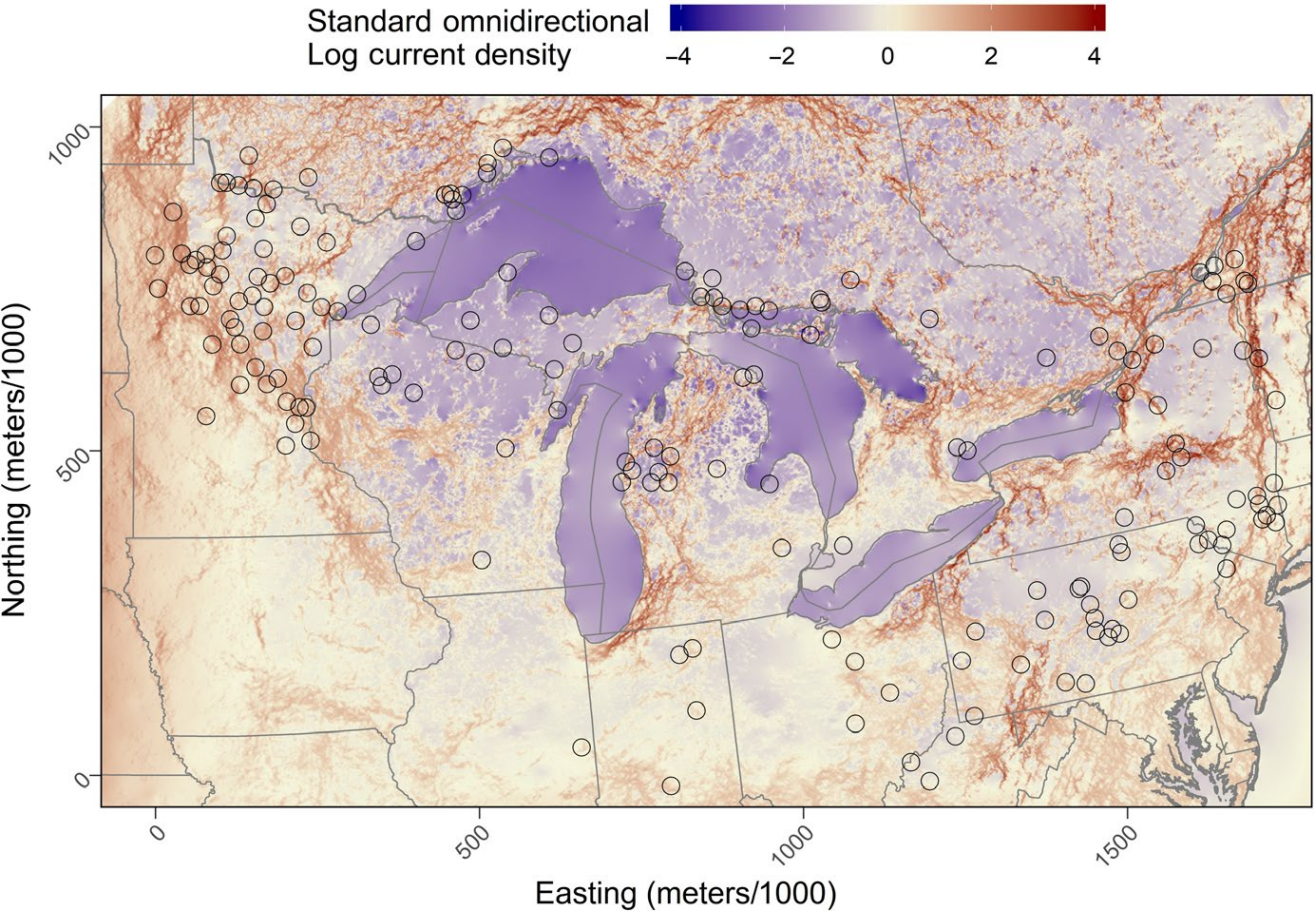
Current density for gene flow through the Great Lakes region. Current density was estimated from the pairwise current of 100 nodes placed on the extremity of all sides of the study area. We used the average standard resistance surface of the top model and rescaled the values from 1 to 100 and used Circuitscape to calculate the cumulative current density of the pairwise iteration of the 100 nodes. We than natural log‐transformed, standardized, and scaled the current density to the mean. A value of 0 indicates areas that have average log‐transformed current density, and value below and above indicate below and above average log‐transformed current density

There were generally spatial patterns of resistance and current density over our study area. For instance, resistance was high in the lower Great Lake states, but there was a zone of low resistance that overlapped Pennsylvania and Ohio (Figure 5). Conversely, resistance was lower in the upper Great Lake States and farther south. Though resistance seemed high in New York State, there was an “L”‐shaped corridor of low resistance and high current that followed the border between Vermont and New York State from the border of Quebec and turned west from the tristate boundary and continued all the way to the Canada and US border between Lake Ontario and Lake Erie. This area with low resistance connected QC, VT, and NY State with a square corridor around the Adirondack (Figure 6). This corridor also connected to southern Ontario and jumped the St. Lawrence River from the Thousand Islands Archipelago between Canada and the United States into Ontario.

On the Lower Peninsula of Michigan (LPM), resistance was low and current was high compared with the Upper Peninsula. The high resistance and low current area of the UPM also overlapped to some extent northern Wisconsin and northeastern Minnesota. In southern Ontario, Canada, resistance was high and decreased into central Ontario but increased once again toward northeastern Ontario. The current mimicked this pattern but was also amplified on the Niagara Escarpment. On the shores of Lake, Huron resistance was low and current high, and this was also true for Manitoulin Island. Comparatively, northwestern Ontario had low resistance and consequently high current. Other important corridors were the high current areas that followed Mississippi River into Manitoba and northern Ontario and the pinch point that connected Michigan to northern Ontario from the Straits of Mackinac.

## 4 DISCUSSION

We originally hypothesized that gene flow percolated between the Great Lakes and deep snow areas but was also hindered by low forest cover and by the transportation infrastructure. We found significant spatial structure where gene flow was constricted by the Great Lakes and areas with low and high forest cover with deep lake‐effect snow impeded gene flow, while intermediate forest cover facilitated gene flow in the Great Lakes region. Although we did not anticipate the bimodal effect of forest cover, our findings were consistent with our isolation‐by‐resistance hypothesis (*H*_2_).

The warming climate will only aid in the expansion of vagile habitat generalists, since areas of deep lake‐effect snow may eventually disappear. However, the Great Lakes and areas with low forest cover limited gene flow more than snow; therefore, we can only predict that less mobile and generalist species will have a more difficult time spreading northward across this landscape as they track climate. In addition, connectivity will be further restricted if these species are not resilient to disturbance such as road, highways, and urban development. Our results are in accordance with a previous analysis that estimated the margin of success of species ability to track climate between natural regions across the United States. The authors found that the network of habitat patches of the Great Lakes region largely failed at connecting habitat that species might use to track the warming climate (McGuire et al., 2016).

We also predicted that certain pathways connected populations throughout the region (Figure 1). We wrongly expected the Upper Peninsula of Michigan to be a main pathway to northern Ontario but found instead that the Lower Peninsula was more probable. This would mean that species would be forced to cross the Straits of Mackinac and the St. Marys River. The route from the Lower Peninsula is likely difficult for many species, since it requires the crossing of a 5‐km stretch of water, or ice in the winter months. The latter will become less common as the climate warms. It is also likely that the UPM will be favored as climate warms, since deep lake‐effect snow will become less likely and this route also does not require crossing a 5‐km stretch of water. However, both the LPM and UPM require the crossing of the St. Marys River. We also incorrectly expected that the Niagara region between Lake Ontario and Erie would be an unimportant route. However, the Niagara region might only be traversable by mobile species such as the bobcat that are resilient to anthropogenic disturbances, since the area has a high density of roads compared with other pathways through the Great Lakes (Figure 3c). For example, gene flow of the highly adaptable raccoon (*Procyon lotor*) was restricted through this route between Canada and the United States and this also matched the pattern of raccoon rabies incidences at the time (Cullingham, Kyle, Pond, Rees, & White, 2009).

### 4.1 Natural barriers

We found that the Great Lakes, on average, had high resistance compared with any other feature on the landscape (Figures 4 and 5). Although at first glance, we did find that the Great Lake barrier itself did not contribute much to the optimized resistance values of the top models, but this was due to the snow layer that created a spatial trend within the Great Lakes (Figure 5). The pattern within the lakes was caused by the large quantity of annual snow received due to the lake effect (Figure 3d). The variability in snow found within the Great Lakes seemed to be important, since models that included snow ranked better than the Great Lakes and forest cover models (Table 1). For example, in Keweenaw Bay in Lake Superior (KB; Figure 5), resistance was higher than the rest of the lake and this was due to the high amount of snow that the bay received annually due to the lake effect (Figure 3d).

In all, even if the Great Lakes and annual snow were confounded, the outcome was the same, the Great Lakes were without a doubt a barrier to gene flow whether it was caused by the lakes themselves as a barrier or the deep snow that accumulated on them in winter when they freeze or both. On land, snow alone did not seem to be quite important overall, but there were a few areas where the lake‐effect snow was quite important, this was the UPM, the Bruce Peninsula, and the area to the east of Lake Ontario that intersected some parts of the Adirondacks (Figure 3d). However, only the UPM and the east side of Lake Ontario had higher resistance (Figure 5). Our prediction that snow restricted gene flow northward over our study area did not hold, this could have been due to the low number of samples in the more northern areas of the bobcat's distribution where snowfall is much higher (Figure 3d).

### 4.2 Gene flow into the northern range limit

Gene flow in southern Ontario was more likely through the Niagara region from New York State, since we found that the land that connected both areas was more resistant to gene flow and this was due to low forest cover (Figures 3b and 4). From this point, gene flow was possible into central Ontario, since resistance decreased and current increased northward. Bobcat have been reported in central Ontario in the past, and occurrences are more common than in southern Ontario. In fact, bobcats were once common on the Bruce Peninsula (de Vos, 1964).

Gene flow to northern Ontario was not facilitated by the UPM and the forests of western Ontario as we previously hypothesized. We found that gene flow into northern Ontario through the UPM is less likely and had more likely occurred through the LPM (Figures 5, 6). This was a surprising result, since the UPM previously seemed more likely because of the high amount of forest cover and because the LPM was an area where the bobcat was slowly recolonizing after it was extirpated (Figure 2). However, considering our results the LPM is more appropriate because of the intermediate amount of forest cover which seemed to amplify gene flow (Figures 4 and 5). In addition, snowfall on the UPM was much higher than the LPM. In fact, the average annual snowfall on some areas of the UPM exceed well over 6 meters (Figure 3d). In contrast, the average annual snow on the LPM was shallower with depths not exceeding 4 meters, but compared to the UPM, these snowy areas occupied less of the land. Furthermore, the major thruway from the LPM to northern Ontario was across the 5.6‐km‐long Straits of Mackinac, which was only feasible in the winter when the lake was frozen. From our own experience tracking bobcats, it is not uncommon to observe bobcat that cross large bodies of water in winter. A bobcat with a GPS collar from our study crossed the North Channel to the Grant Islands, a distance > 5 km across Lake Huron ice (unpublished data).

### 4.3 The importance of intermediate forest cover

Even if the Great Lakes had the highest resistance, forest cover had the highest average contribution to restricting gene flow. Though we previously thought that high tree cover would amplify gene flow, we found that intermediate amount of forest amplified gene flow. Also, forests with 80% tree cover hindered gene flow more than areas with no tree cover (Figure 4). One common assumption is that the bobcat is a habitat generalist (Anderson & Lovallo, 2003); consequently, it does not specialize on any specific habitat type across its range, and therefore, it may perform better in environments with an average amount of forest cover compared with area that are 0 or 80% forest cover. Areas with low forest cover are either urban centers or areas that are predominantly used for agricultural purposes. These areas are mostly found in the Midwest corn belt of the United States where tree cover was low over our study area (Figure 3b). The corn belt was first cleared for agriculture in the 1850s and since then has been an area with low biodiversity and intensive agriculture use (Jenkins, Houtan, Pimm, & Sexton, 2015; Nassauer, Santelmann, & Scavia, 2007). Compared with more forested areas, the corn belt may have lower abundance and diversity of prey species that may not be able to sustain the bobcat.

On the other extreme, areas with high forest cover were areas where bobcat gene flow was obstructed, these forests were generally found in the upper Great Lakes region and were also found in the Appalachian corridor and the Adirondacks. Some of these forests with high amount of canopy cover also had high annual snowfall compared with other areas of the bobcat range in our study area (Figure 3b and d). Bobcat may not be able to effectively hunt in dense forests with high annual snowfall compared with forest with similar snow and intermediate forest cover.

The bobcat needs some forest cover to stalk prey (McCord, 1974), but perhaps the forest cover cannot be so dense as to reduce visibility and muffle sound, since the bobcat relies heavily on sight and sound to hunt (McCord & Cardoza, 1982); therefore, forests with an intermediate amount of cover might be more preferred by bobcat. The ability to see and catch prey is a function of forest cover, but also, there is an interplay with snow depth, since deep snow reduces their ability to hunt (McCord, 1974). To some extent, forest cover was also associated with road density, in cases where road density is high, and forest cover was reduced, and this happened near urban areas (Figure 3b–c). In more rural communities, where forest cover is intermediate with road density, bobcat gene flow was amplified.

### 4.4 Bobcat range expansion

In general, our results suggest that the northward expansion of the bobcat in the Great Lakes region has been facilitated by intermediate forest cover. Therefore, the expansion of the bobcat is in part a response to the decrease in forest cover due to land clearing and forestry in the Great Lakes region. In fact, in the northern Great Lakes US states the area occupied by open land has increased from 12.3% to 41.3% since 1836 (Schulte, Mladenoff, Crow, Merrick, & Cleland, 2007), which is within the range of optimal forest cover for bobcat gene flow that we found in our analysis (Figure 4). This land clearing may have opened previously unavailable habitat in northern Ontario to bobcat, but deep winter snow may have still been a limiting factor until snow depth subsided due to climate change in later years (Dyer & Mote, 2006).

After this point, further north, bobcat began to be harvested by trappers in northwestern Ontario in the early 1900s and in northeastern Ontario in the mid‐1900s (de Vos, 1964). The 50‐year lag period between these two areas could have been due to the disparity in the amount of annual snow received in both areas (Figure 3d). Northwestern shores of Lake Superior received less snow than the northern shores of Lake Huron. Currently, on the north shores of Lake Huron intermediate forest cover is still important to bobcat, since bobcat in this area are almost exclusively found in rural communities within 50 km of urban centers (Marrotte et al., In Review). The increasing density of human disturbances such as roads, rail lines, urban areas, rangeland, and agricultural land would have also further amplified colonization, because road plowing and snow compaction would have become more frequent, which allowed bobcat to move around and hunt more effectively. Therefore, at their northern limit, areas with intermediate forest cover that have an intermediate density of roads may have mediated the colonization of bobcats into areas the bobcat generally would not have occupied, because of deep annual snow.

Overall, land use and cover change and the decreasing snow pack due to climate change will only facilitate the expansion of bobcat and we can only expect to find bobcats farther north each year. However, it is important to note that the landscape of southern Ontario has impeded gene flow and consequently movement of bobcat over the past decades. As the climate continues to warm and species are tracking their bioclimatic niche through the Great Lakes region, we can only expect that less mobile species are less likely to cross southern Ontario. Other routes are already blocked by natural features such as the St. Lawrence River, the 5.6‐km‐long Straits of Mackinac, and the St Marys River, and the additive effect of human modification will undoubtedly further restrict these routes and reduce future potential biodiversity.

## CONFLICT OF INTEREST

None Declared.

## AUTHOR CONTRIBUTIONS

RRM and JB conceived the study. RRM, JB, and PJW collected the samples. RRM wrote code, ran simulations, analyzed the data, and wrote the manuscript. JB and PJW critically reviewed the manuscript.

## Data Availability

The data and R scripts that support the findings of this study are openly available on Dryad at https://doi.org/10.5061/dryad.zgmsbcc6d.
